# The New Derbyshire Royal Infirmary

**Published:** 1894-07-14

**Authors:** 


### HOSPITAL CONSTRUCTION.

### THE DERBYSHIRE ROYAL INFIRMARY.

The opening of the Derbyshire Royal Infirmary on Saturday was a complete and signal success. The arrangements reflected the greatest credit on Mr. Henry Boden, J.P., the President, the committees in charge of them, and the honorary and resident staff. More than a thousand people paid five shillings each to be present at the ceremony, which was characterised throughout by great enthusiasm. The list of speakers was long, and included the President, the Duke of Devonshire, Mr. Victor Cavendish, M.P., the High Sheriff, Sir Douglas Galton, Dr. Edward Seaton, Sir H. Wilmot, and Mr. Burdett.

It is, we believe, a unique circumstance that the whole proceedings, despite the number of speakers, were over in less than an hour and a half, that every speaker was well and distinctly heard, and that the speeches were brief, terse, and to the point. We have dealt with the special liberality of the inhabitants of the city and county of Derby elsewhere, and shall confine our remarks here to a description of the new buildings, which may be regarded as the most perfect specimen of a county hospital yet erected in this country. Great credit is due to the architects, Messrs. Young and Hall, Southampton Street, Bloomsbury, who have made a study of hospital architecture, and who were chosen for the work by the committee after careful inspection of certain selected hospitals recently built. In view of the lamentable failure which has characterised the selection of plans by architect assessors in recent competitions, we earnestly hope that in future hospital and asylum committees will cease to apply to the Council of the Royal Institute of British Architects when they wish to select an assessor, unless or until that body exhibit a more intelligent regard to the proprieties and requirements of these competitions than they have heretofore displayed. Experience proves that a limited, or any other competition, will not secure the erection of the best possible building, unless the architect assessor has associated with him someone who has a sound knowledge of hospital administration and the purposes to which each particular building, when completed, will be put.

These remarks do but emphasise the wisdom, and so add to the credit, which ought properly to devolve upon the committee at Derby. They have struck out a line for themselves, and so have secured a hospital which will compare favourably with any existing institution of the kind. The following approximate financial statement cannot fail to be of interest as showing the vastness of the undertaking, and the public spirit which has marked the proceedings from first to last. The liberality of Derby and Derbyshire ought to stimulate contributions to hospitals everywhere throughout the United Kingdom.

Approximate Financial Statement.

| | £ | s. | d. | £ | s. | d. | £ | s. | d. |
|---|---|---|---|---|---|---|---|---|---|
| Amount to which the Governors are already committed ... ... | | | | | | | 94,400 | 0 | 0 |
| Further expenditure necessary: | | | | | | | | | |
| Making roads, footpaths, asphalting, &c. ... ... ... | 1,434 | 0 | 0 | | | | | | |
| Levelling, forming, soiling, and seeding grass spaces, &c. ... ... ... | 888 | 0 | 0 | | | | | | |
| Trees, shrubs, &c., and planting ... | 253 | 0 | 0 | | | | | | |
| | | | | 2,575 | 0 | 0 | | | |
| Electric light in four large and four small wards ... ... | | | | 300 | 0 | 0 | | | |
| Eye hut ... ... ... ... ... | | | | 600 | 0 | 0 | | | |
| Digging up old drains, say approximately ... ... | | | | 1,000 | 0 | 0 | | | |
| | | | | | | | 4,475 | 0 | 0 |
| To meet the expenditure the following sums have been promised: | | | | | | | 98,875 | 0 | 0 |
| Donations to Building Fund to date, including workmen's donations and Mayor's Auxiliary Fund ... ... | | | | 64,933 | 0 | 0 | | | |
| Donation from Mr. Walter Evans for Evans Block ... | | | | 12,000 | 0 | 0 | | | |
| Donation from Mr. G. H. Strutt for Strutt Block ... ... | | | | 12,000 | 0 | 0 | | | |
| | | | | | | | 88,933 | 0 | 0 |
| Amount still to be raised ... | | | | | | | £9,942 | 0 | 0 |

[We intended to publish a full description of the buildings, with plans, but as the blocks have not come to hand we are obliged to hold over both till next week.]

